# A Decade of Sustaining Best Practices for Tobacco Control: Indiana's Story

**DOI:** 10.5888/pcd9.110144

**Published:** 2012-01-12

**Authors:** Stephen J. Jay, Mohammad R. Torabi, Miranda H. Spitznagle

**Affiliations:** Indiana University School of Medicine, Department of Public Health; Department of Applied Health Science, Indiana University, Bloomington, Indiana; Tobacco Prevention and Cessation Commission, Indiana State Department of Health, Indianapolis, Indiana

## Abstract

The Indiana Tobacco Prevention and Cessation Agency (ITPC) was created in 2000 to address high tobacco use rates. This independent state agency, using Centers for Disease Control and Prevention (CDC) Best Practices for Comprehensive Tobacco Control Programs, administered a comprehensive program that supported community health coalitions and evidence-based public policy changes. From 2000 to 2011, ITPC operated in difficult budgetary and political environments and with less than 20% of the funding recommended by CDC. ITPC and its partners enabled social and cultural changes, reduced cigarette use rates, and increased the number of community smoke-free environments. Public health leaders in Indiana agreed that the independent agency model was effective in reducing the costs associated with tobacco-use-related disease and death. Despite broad public support for ITPC and its work, on April 29, 2011, the Indiana legislature passed a controversial budget bill that abolished the ITPC executive board and transferred its budget and function to the Indiana State Department of Health (ISDH). Although the tobacco control program is not insulated from political interference, the ISDH commissioner has created a new Tobacco Prevention and Cessation Commission, whose members report directly to him, with commitment to continue the programmatic focus of the former ITPC. Restoring full funding to the tobacco control program is necessary if Indiana's goal of decreasing the health care and business costs of tobacco use-related diseases are to be achieved.

## Introduction

In 2000, the Indiana Tobacco Prevention and Cessation Agency (ITPC), an independent state agency created to decrease the state's burden of tobacco use, implemented and evaluated a state program created by statute on the basis of the Centers for Disease Control and Prevention's (CDC's) Best Practices for Comprehensive Tobacco Control Programs (Best Practices) (1,2). When ITPC was organized in 2000, 27% of Indiana adults smoked, compared with the national median of 23% (3). In 2010 in Indiana, 9,700 deaths and $4.7 billion in annual health and other economic costs, including $487 million in Medicaid payments, were attributed to tobacco use (4,5). Although progress has been made in tobacco control during the past decade, Indiana still has a high prevalence of tobacco-use-related addiction and disease, which is partly because of Indiana's historically low investments in public health and tobacco control; the state ranks 29th among states in funding programs to prevent children from smoking and help smokers quit and 49th in per capita public health spending (6,7).

We describe in this article the creation of ITPC, its implementation of evidenced-based interventions, and the health and economic results of policy changes effected by ITPC from 2000 to 2011. ITPC encountered funding and political challenges throughout its 10-year history and was abolished by the Indiana legislature in 2011. We review the entity that replaced ITPC, the Indiana State Department of Health (ISDH) Tobacco Prevention and Cessation Commission (TPCC), as well as the current threats and opportunities for Indiana's tobacco control programs.

## Creation of the Indiana Tobacco Prevention and Cessation Agency

Indiana was 1 of 46 states that settled with the tobacco companies in 1998 to receive more than $4.5 billion for the first 25 years and additional monies, in perpetuity, through the Tobacco Master Settlement Agreement (MSA) (8). State lawmakers recognized the need to identify an appropriate entity to manage the state's tobacco prevention funding and programs (9).

The ISDH implemented the national American Stop Smoking Intervention Study (ASSIST) from 1991 to 1999, with little success. Indiana was 1 of only 2 ASSIST states that experienced an increase in tobacco consumption during the study period, which was attributed to Indiana's weak public health infrastructure (10).

The basic structure of the new board that was to oversee the statewide cessation programs was very important to me. I wanted an autonomous group of people deciding how best to deliver the cessation effort. Up to that time, Indiana was fragmented in the delivery of anti-smoking programs. The Board of Health, Department of Mental Health, the superintendent of Public Instruction and other state agencies were attempting to create competing programs. I was adamant that there be one unified approach. Representative Brown and I both strongly agreed that this operation would be free from outside political influences.

Lawrence M. Borst, Indiana State Senator (9)

State leader Sen. Larry Borst drew on the experiences from other states to develop an independent tobacco control entity (9). Two of the four states that independently settled their lawsuits against the tobacco companies created foundations to manage their state tobacco control programs. Later, states, including Ohio and Minnesota, established separate foundations to oversee the programs.

Indiana lawmakers chose to create an independent, single-purpose state agency to coordinate programs to reduce tobacco use. Policy makers designed ITPC to create synergy among 5 state agencies that already had some role in tobacco use prevention, to increase competitiveness for national grants and contracts and to reduce the risk for political interference by the tobacco industry (1).

Senate Enrolled Act 108, signed into law in 2000 by Governor Frank O'Bannon, created the ITPC Executive Board (1). In 2000, CDC's recommended annual funding for Indiana was $35 to $97 million; lawmakers appropriated $35 million, placing Indiana in a national leadership position as 1 of only 6 states to reach the level of funding recommended by CDC (2). With additional appropriations of $5 million in 2002 and $25 million in 2003, the ITPC board established a 2-year, $32.5 million annual budget and launched the program statewide (11).

## Implementing Evidence-Based Tobacco Control

Since the 1980s, decreases in smoking prevalence in the United States have been attributed to tobacco control interventions, including price increases, comprehensive statewide smoke-free air laws, and a culture supportive of tobacco-free norms. These advances have been supported by comprehensive state tobacco control programs and the implementation of evidence-based interventions (2).

Best Practices is the key guidance document for establishing comprehensive, evidence-based programs (2), and the ITPC executive board adopted it in 2000 as the basis for its comprehensive approach to tobacco control in Indiana (1). Best Practices includes community-based programs, public education campaigns (counter-marketing), cessation interventions, surveillance, and evaluation.

During 2002, ITPC established a statewide network of community-based grantees. Each of Indiana's 92 counties received a grant to support a community-based tobacco control coalition and a minority-based coalition in counties with the largest minority populations (11). The ITPC community-based program allowed communities to choose the lead agency that was best suited for their needs, which led to a diverse mixture of local lead agencies, ranging from hospitals and community-based organizations to universities and local health departments (11). In 2011, because of funding cuts, only 65 counties in Indiana had maintained a local coalition that educated the community about tobacco use, provided prevention and cessation resources, and raised awareness about policies that reduce the burden of tobacco use (12).

ITPC, and now the newly created TPCC, support coalitions and communities in focusing on improving community health by changing smoke-free public policies. Community-based programs participate in frequent education and training opportunities in tobacco control and are provided with ongoing technical assistance (12). ITPC created a statewide public education campaign to increase awareness of the dangers of tobacco use and economic burdens of tobacco use on all citizens (11). This campaign increased public support for smoke-free workplaces from 68% in 2002 to 93% in 2008 (13).

With its tobacco control partners, ITPC developed a program to promote the use of evidence-based interventions to encourage tobacco use cessation. This program includes health provider training, business outreach, and changes in health care settings to ensure that tobacco users have access to tobacco-dependence treatment, including benefits for Medicaid members (12). Since the Indiana Tobacco Quitline (1-800-QUIT-NOW) was implemented in 2006, it has served more than 68,000 Indiana citizens (12).

## Effect of Policy Changes

ITPC and its partners have provided leadership for communities to implement policies that change behaviors. Smoking among adults has decreased by 23%, from 27.4% in 2001 to 21.2% in 2010. (3) Per capita cigarette consumption has decreased by 41% from 2000 to 2010, from 121.4 packs per year to 71.7 packs per year (12). Between 2000 and 2010, high school youth smoking rates have decreased by 45%, from 31.6% to 17.5% (14).

**Figure 1.**
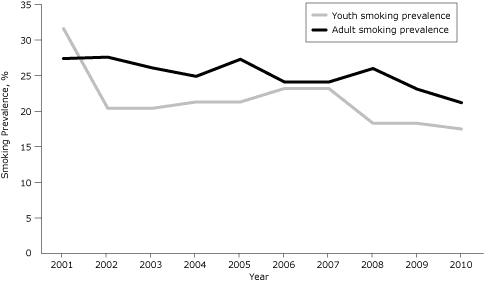
Smoking prevalence in Indiana among adults and youth, 2001-2010. Adult smoking prevalence rates from the Behavioral Risk Factor Surveillance System (3), and youth smoking prevalence rates from the Indiana Youth Tobacco Survey (4,14).

**Table:** 

**Year**	**Smoking Prevalence**	
	**Adults, %**	**Youth, %**
**2001**	27.4	31.6
**2002**	27.6	20.4
**2003**	26.1	20.4
**2004**	24.9	21.3
**2005**	27.3	21.3
**2006**	24.1	23.2
**2007**	24.1	23.2
**2008**	26.0	18.3
**2009**	23.1	18.3
**2010**	21.2	17.5

**Figure 2.**
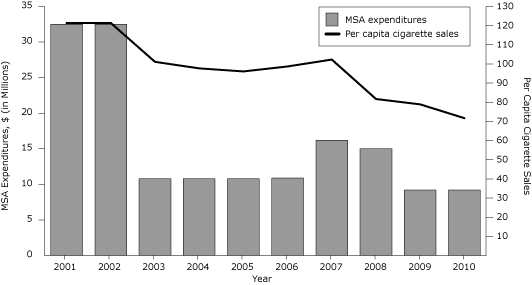
Indiana Tobacco Prevention and Cessation Agency MSA expenditures (11,12) and per capita cigarette sales (12), Indiana, 2001-2010. Abbreviation: MSA, Master Settlement Agreement of 1998.

**Table:** 

** Year**	**MSA Expenditures, $ (Millions)**	**Per Capita Cigarette Sales**
**2001**	32.5	121.4
**2002**	32.5	121.4
**2003**	10.8	101.1
**2004**	10.8	97.7
**2005**	10.8	96.1
**2006**	10.9	98.7
**2007**	16.2	102.3
**2008**	15.0	81.7
**2009**	9.2	78.9
**2010**	9.2	71.7

The number of smoke-free air policies increased between 2001 and 2011. In 2001, 87 public school districts protected youth from secondhand smoke through a 100% tobacco-free campus policy, and 1 university had a tobacco-free campus (Table). In 2011, 234 public school districts were tobacco-free, and 35 colleges and universities had a tobacco-free campus. In 2011, 133 hospital and health care facility campuses in Indiana had tobacco-free grounds policies, compared with none in 2001. In 2001, no local community comprehensive smoke-free air ordinances existed; in 2011, 33 local smoke-free workplace and restaurant ordinances were in place that protect 36% of the state's approximately 6 million people from secondhand smoke exposure (12). (In 2011, in total, 41 local smoke-free air ordinances existed in Indiana; however, 8 had ineffective policy provisions, as defined by a 2006 report of the US Surgeon General [15]).

In 2001, Indiana's cigarette tax was 15.5 cents, compared with the 2001 all-state average of 43.4 cents (16). Indiana's cigarette tax was increased in 2002 and 2007 to its current rate of 99.5 cents per pack. ITPC, working with state health commissioners and statewide health coalitions, helped ensure the passing of the 2002 and 2007 tax increases. These additional tax revenues were then used to promote the Indiana Tobacco Quitline, which encourages smokers to make quit attempts.

Through its state training plan, ITPC built skills in media and policy advocacy among state and local organizations, increasing the capacity to implement social and environmental changes. From 2002 to 2010, Indiana's news media increased coverage and support for tobacco control, generating more than 27,000 news items. Analysis of news media content showed that regional newspaper coverage of tobacco taxes, smoke-free air policies, and effect of smoking on health care costs increased youths' belief that youth smoking is an important health issue (17).

ITPC has promoted public-private partnerships to improve health policies. In 2004, leaders from all major hospitals in central Indiana implemented tobacco-free policies for their campuses; these leaders were instrumental in passing Indianapolis's first smoke-free workplace law. In 2009, they also urged Indianapolis law makers to strengthen the state's smoke-free workplace law to include all workplaces (18). Throughout Indiana, similar collaboration and partnerships have been developed to change social norms and public policy. An evaluation conducted by Washington University in St. Louis, Missouri, on how state tobacco control programs implemented CDC's Best Practices found that Indiana's integration of its tobacco control partners' network was highly efficient in serving information needs of partners (19).

## Success Despite the Challenges

Despite chronic budget pressures and interference by the tobacco industry, ITPC continued to implement comprehensive tobacco control consistent with Best Practices and emerging research (18). Although the 2002-2003 annual budget of $32.5 million was near the CDC-recommended minimum, funding cuts occurred in 2004-2005 and 2010-2011 (18), and programs and services decreased accordingly (Figure 2). ITPC's community and statewide grants and counter-marketing programs experienced budget decreases. Community and statewide grants decreased from 2002 to 2010 by 44%, and counter-marketing programs were cut by 80%. (11,12) With each state budget cycle, there has been pressure to limit the agency's activities and funding, except during 2008-2009, when ITPC was appropriated a $5.4 million increase in funding, a portion from the state cigarette tax increase. This increase was reduced in the next biennium by $5 million (18).

The role of the tobacco industry in promoting destabilization or elimination of tobacco control programs has been well documented in Indiana and in other states (18,20). From 2000 to 2009, the industry spent more than $4 million on lobbying in Indiana. From 1997 to 2008, tobacco companies contributed $560,884 to elected officials, many of whom were high-ranking leaders in government (18).

In 2010, efforts by the Indiana General Assembly to eliminate the ITPC executive board's authorizing legislation, abolish the agency, and move the program to the ISDH jeopardized the tobacco control program's future (18). The stated reason for these actions was to avoid duplication of ITPC and ISDH efforts, a concern not validated by public health experts (18). The next year, 19 days before the end of the 2011 legislative session and without a public hearing, the state's budget bill (HB 1001) was amended by the chair of Senate Appropriations Committee to eliminate ITPC (18). Despite the extensive opposition to this proposal by public health and tobacco control advocates and the media statewide, the Indiana state legislature authorized the following action: "The Indiana tobacco use prevention and cessation executive board is abolished July 1, 2011, [and] all assets, obligations, powers, and duties" are to be transferred to ISDH (18,21,22).

## Looking to the Next 10 Years

After HB 1001 was signed on May 10, 2011, by Governor Mitch Daniels, the State Health Commissioner and former ITPC executive director jointly announced to state tobacco control advocates that the ITPC programs would move ISDH to the newly created TPCC (written communication, G.N. Larkin and K.S. Sneegas, May 2011).

Details of the structure and function of this tobacco control program await clarification. A potential positive effect of this new commission includes more emphasis on tobacco control interventions among all areas of health in ISDH. However, this new structure is more vulnerable to political interference and influence from the tobacco industry. In such an environment, with inevitable threats to its budget, the TPCC may find it increasingly difficult to sustain effective Best Practices in Indiana (18).

For the TPCC to accomplish its current statewide tobacco use reduction goals using Best Practices, the following conditions need to be met: Indiana receives funding at the level recommended by CDC for all 5 components of the Best Practices model; Indiana's statewide cessation system, which includes the Indiana Tobacco Quitline, expands and is coupled with effective public education campaigns; Indiana enacts a comprehensive smoke-free workplace law that includes restaurants and bars; in the absence of a state law, local communities proceed with local ordinances to protect citizens from exposure to secondhand smoke; and taxes for cigarettes and other tobacco products increase to reflect national trends and reduce the state's tobacco-related disease burden and other economic costs.

The challenge of ISDH is to consolidate the support of the public and of policy makers in achieving short-term goals of a statewide smoke-free air law and an increase in the cigarette tax. Accomplishing these goals will increase the likelihood that Indiana's tobacco control program can address long-term goals of expanding its programs to achieve *Healthy People 2020* tobacco use objectives (23).

A further challenge for ISDH is to ensure stable and increased program funding over time for the expansion of Best Practices. Science has shown that investment in tobacco control produces major returns on investment. The more states spend on tobacco control programs, the larger the decrease in adult smoking, even when controlling for other factors such as high tobacco prices (24). Such returns on investment would have a beneficial effect on Indiana's fiscal health. For example, if Indiana had been investing $35 million annually in tobacco use prevention since ITPC's inception, the state could have reduced tobacco-use-related costs by $479 million, $78 per capita, by reducing adult smoking (24).

Many of the long-term objectives to decrease adult and youth smoking, established by the ITPC executive board in 2000, have been achieved. The test of the next decade will be to build on these successes, which requires continued strong program leadership and a focus on sustaining evidence-based tobacco control programs, as well as public support and political will of policy makers to make informed decisions that support the health and economic interests of Indiana citizens.

## Figures and Tables

**Table.** Comparison of Indiana's Smoke-Free Air Policy, by Location, 2001 and 2011

Location	No. in 2001	No. in 2011^a^
Public school districts with a 100% tobacco-free campus	87	234
Colleges and universities with a tobacco-free campus	1	35
Hospitals and health care facilities with a tobacco-free campus	0	133
Local smoke-free workplace and restaurant ordinances	0	33^b^

Source: Indiana Tobacco Prevention and Cessation Agency (12).

a As of June 30, 2011.

b In total, 41 local smoke-free air ordinances exist in Indiana, but 8 have ineffective policy provisions, as outlined by a 2006 report of the US Surgeon General (15).
